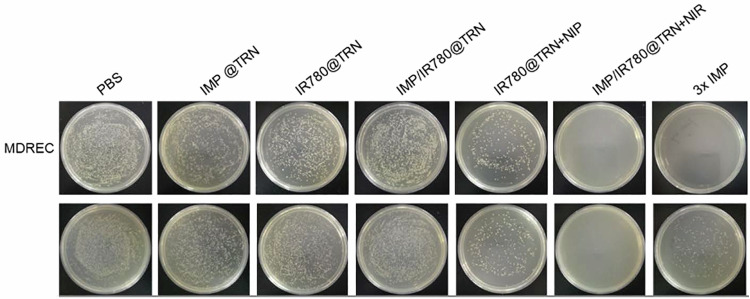# Author Correction: Thermo-responsive triple-function nanotransporter for efficient chemo-photothermal therapy of multidrug-resistant bacterial infection

**DOI:** 10.1038/s41467-025-59414-w

**Published:** 2025-04-29

**Authors:** Guangchao Qing, Xianxian Zhao, Ningqiang Gong, Jing Chen, Xianlei Li, Yaling Gan, Yongchao Wang, Zhen Zhang, Yuxuan Zhang, Weisheng Guo, Yang Luo, Xing-Jie Liang

**Affiliations:** 1 Key Laboratory for Biorheological Science and Technology of Ministry of Education, State and Local Joint Engineering Laboratory for Vascular Implants, College of Bioengineering, Chongqing University, Chongqing, 400044 P. R. China; 2 Chinese Academy of Sciences (CAS) Key Laboratory for Biomedical Effects of Nanomaterials and Nanosafety, CAS Center for Excellence in Nanoscience, National Center for Nanoscience and Technology of China, Beijing, 100190 P. R. China; 3 Department of Materials and Energy, Southwest University, No. 2 Tiansheng Street, Beibei District, Chongqing, 400715 P. R. China; 4 University of Chinese Academy of Sciences, Beijing, 100049 P. R. China; 5 Department of Clinical Laboratory Medicine, Southwest Hospital, Army Medical University, Chongqing, 400038 P. R. China; 6 Translational Medicine Center, Key Laboratory of Molecular Target & Clinical Pharmacology and the State Key Laboratory of Respiratory Disease, School of Pharmaceutical Sciences & The Second Affiliated Hospital, Guangzhou Medical University, Guangzhou, 510260 P.R. China; 7 Nuclear Medicine and Molecular Imaging Key Laboratory of Sichuan Province, Department of Nuclear Medicine, the Affiliated Hospital of Southwest Medical University, Sichuan, 646000 P. R. China

Correction to: *Nature Communications* 10.1038/s41467-019-12313-3, published online 24 September 2019

In the version of the article initially published, due to a mistake during figure preparation, in Fig. 6i, the image of the IMP @ TRN, MDREC plate was a duplicate of the IMP @ TRN, MRSA plate below. The corrected version can be seen as Fig. 1, below. This amendment serves to correct the article.

Fig. 1 Corrected Fig. 6i.