# Supplementary material for: Thermo-responsive triple-function nanotransporter for efficient chemo-photothermal therapy of multidrug-resistant bacterial infection
Source: Nat Commun. 2019 Sep 24;10:4336. doi: 10.1038/s41467-019-12313-3 (PMC6760232; doi:10.1038/s41467-019-12313-3)
Supplement: Supplementary file 1 — Supplementary Information [file 41467_2019_12313_MOESM1_ESM.pdf]

Supplementary Information

Thermo-responsive triple-function nanotransporter for efficient chemo-photothermal therapy of multidrug-resistant bacterial infection

Qing et al.

Supplementary Information

Thermo-responsive triple-function nanotransporter for efficient chemo-photothermal therapy of multidrug-resistant bacterial infection

Guangchao Qing, Xianxian Zhao, Ningqiang Gong, Jing Chen, Xianlei Li, Yaling Gan, Yongchao Wang, Zhen Zhang, Yuxuan Zhang, Weisheng Guo, Yang Luo, Xing-Jie Liang

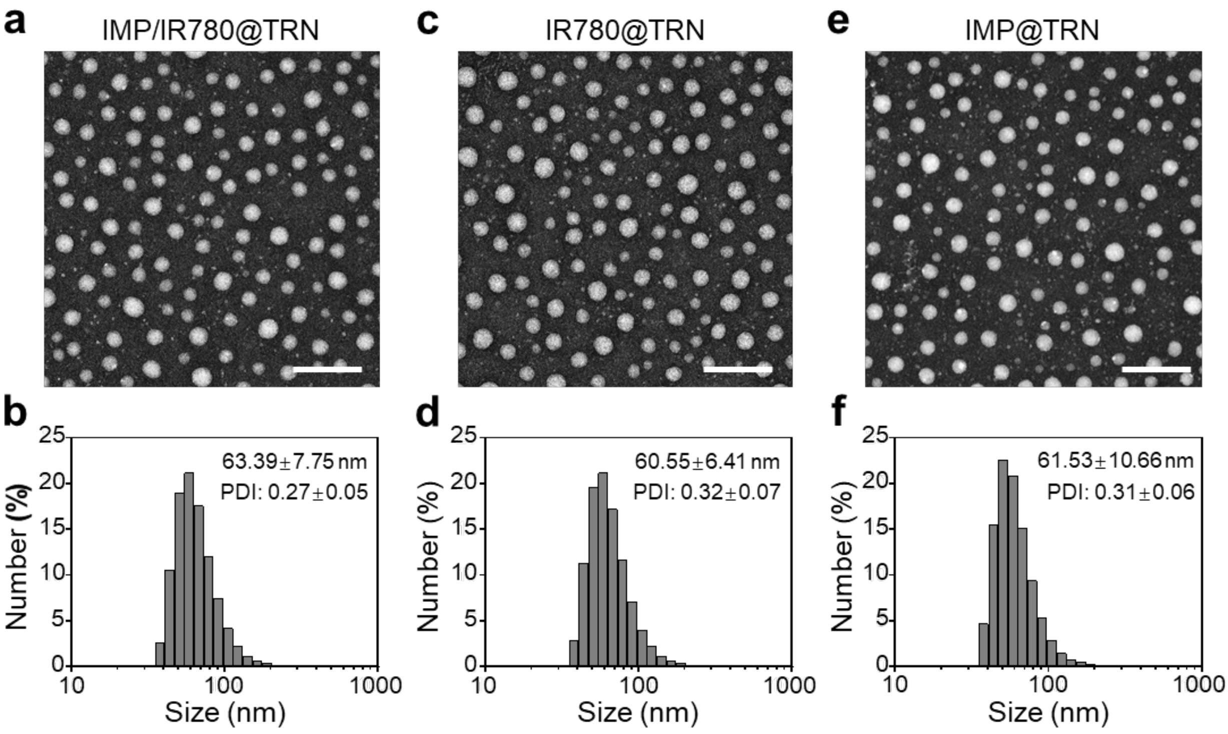

Supplementary Figure 1. Morphology and size of IMP/IR780@TRN, IR780@TRN and IMP@TRN.

a, **b** IMP/IR780@TRN. **c**, **d** IR780@TRN. **e**, **f** IMP@TRN. Data are presented as mean ± s.d. (n=3). Scale bars: 200 nm. Source data are provided as a Source Data file.

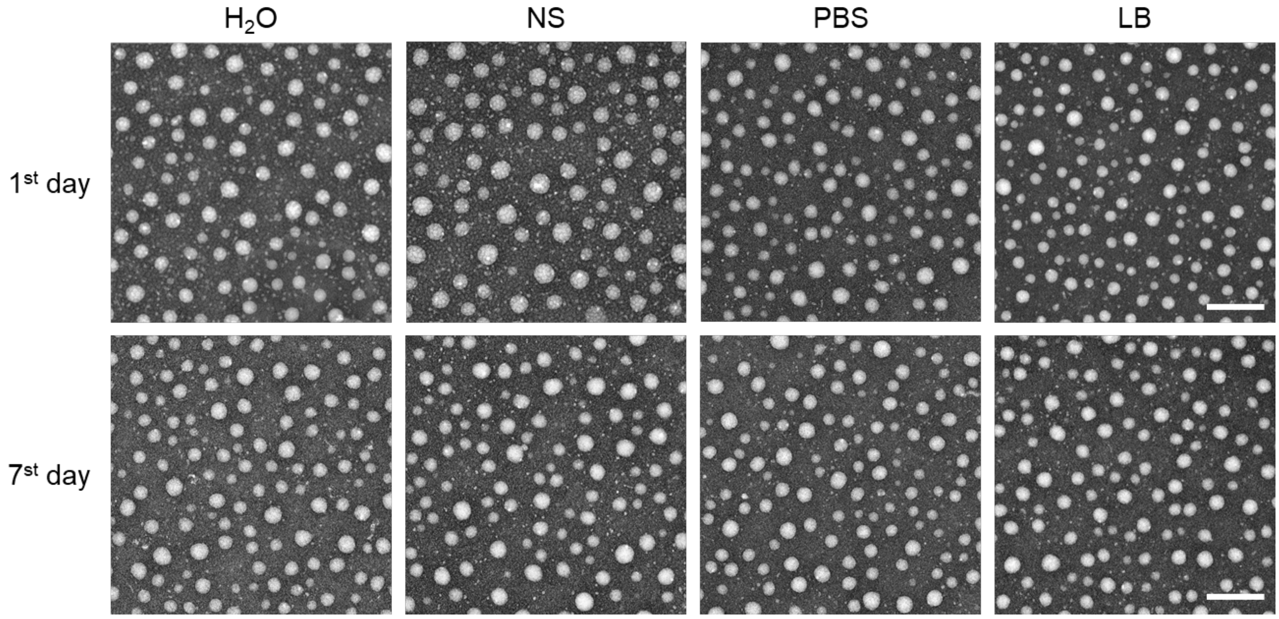

Supplementary Figure 2. TEM images of the TRIDENTs dispersed in four solvents. The size and morphology of the TRIDENTs showed no obvious change during the testing process. Scale bars: 200 nm.

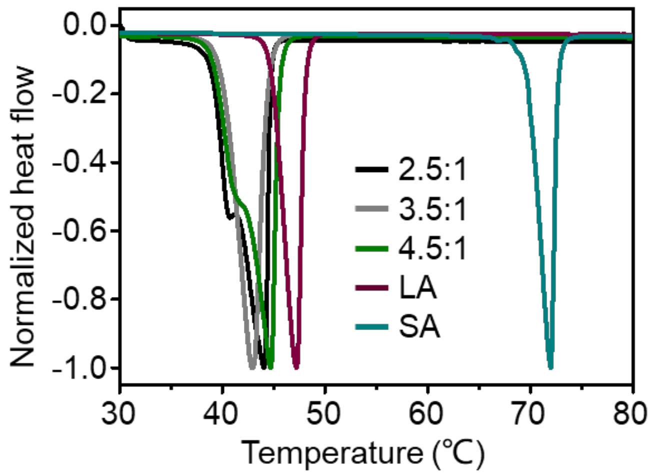

Supplementary Figure 3. DSC curves of the TRNs prepared with different mass ratios of LA/SA.

The phase change temperature of the TRN is lower than any of the components. Source data are provided as a Source Data file.

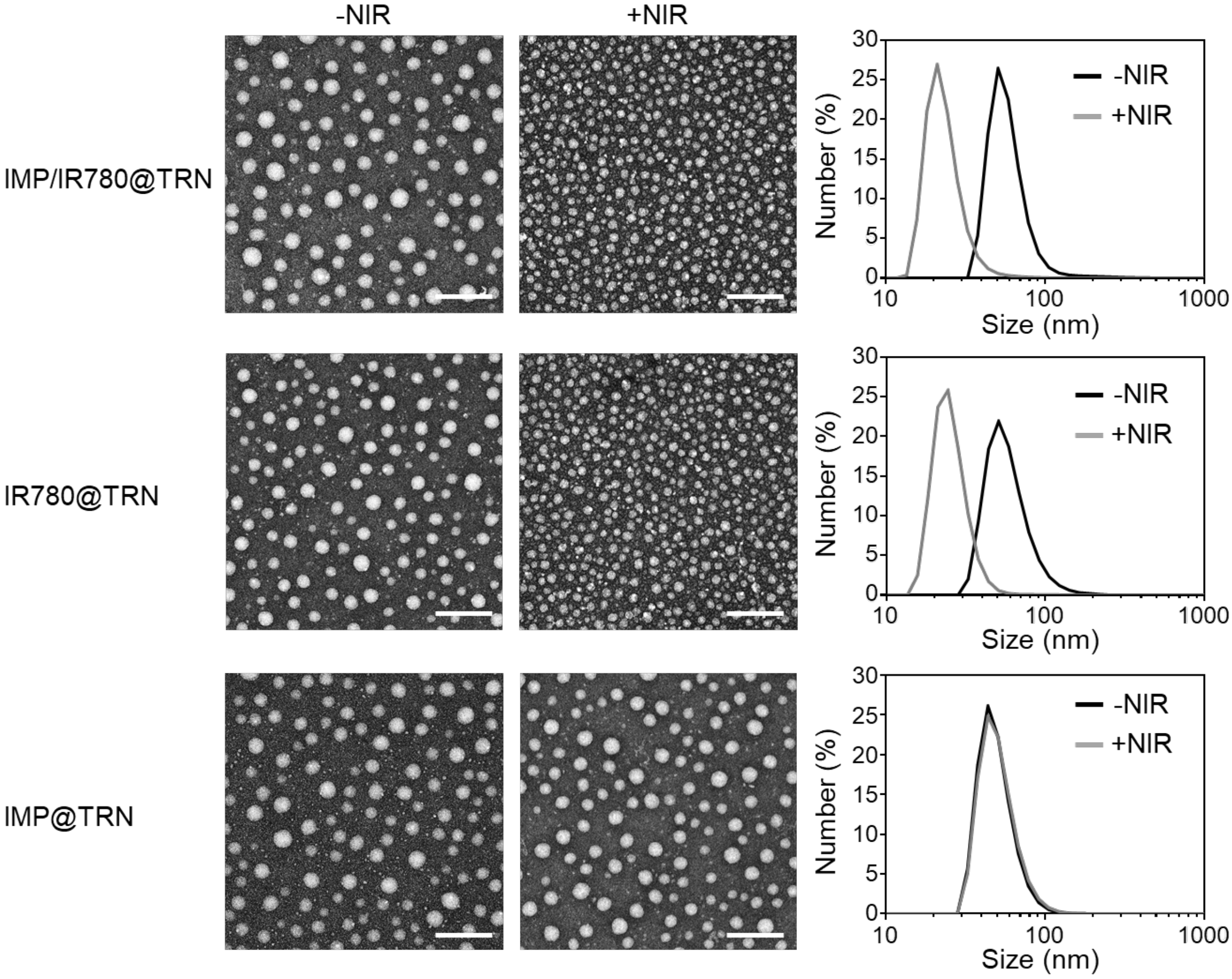

Supplementary Figure 4. Morphological changes of the TRNs before and after NIR irradiation. The size of the IMP/IR780@TRN and IR780@TRN became smaller after the irradiation while no obvious changes were obtained from the IMP@TRN. All TRNs remained spherical. Scale bars: 200 nm. Source data are provided as a Source Data file.

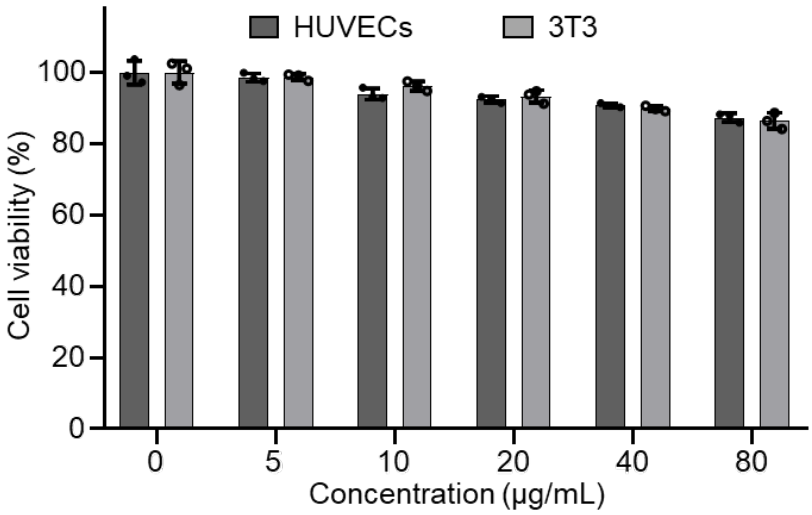

Supplementary Figure 5. Cytotoxicity of the IMP/IR780@TRN to 3T3 cells and HUVECs *in vitro*. The concentration refers to the amount of loaded IMP (loading rate = 51.62±3.68%) and IR780 (loading rate = 74.85±6.60 %). Data are presented as mean ± s.d. (n=3). Source data are provided as a Source Data file.

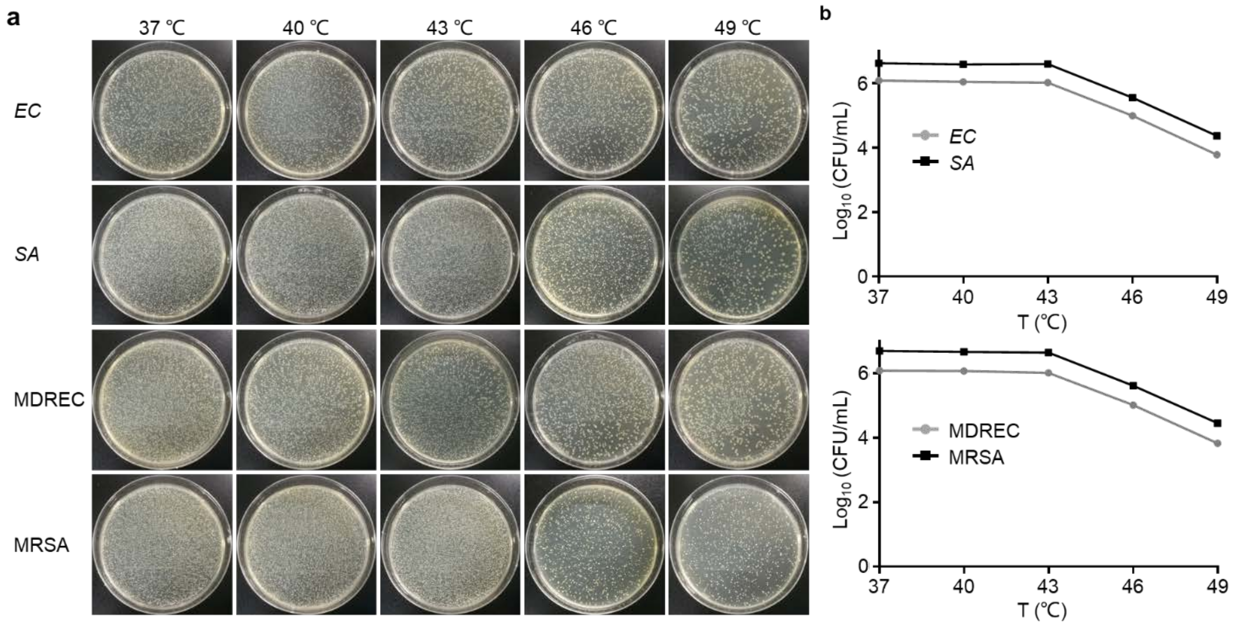

Supplementary Figure 6. Thermal response of the bacteria to various temperatures. a Images of the colonies formed on LB-agar plates. **b** Statistical analysis of the bacterial cell viability by $\log_{10}$ (CFU/mL). Source data are provided as a Source Data file.

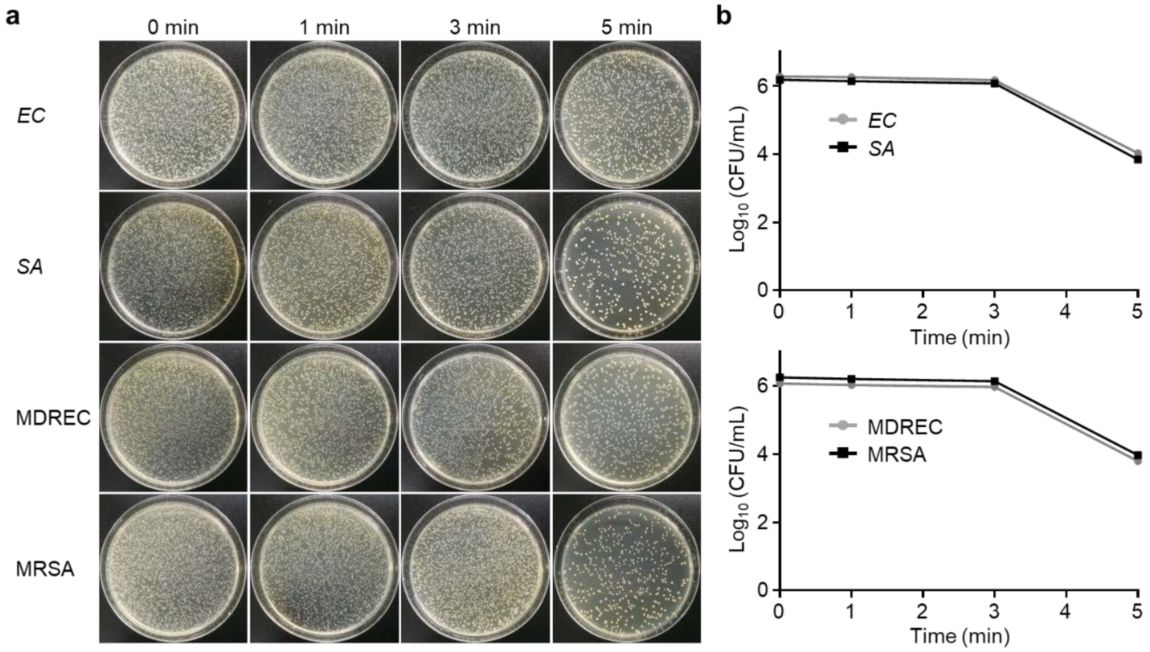

Supplementary Figure 7. Thermal response of the bacteria treated at 49℃ for different time. a Images of the colonies formed on LB-agar plates. **b** Statistical analysis of the bacterial cell viability by $\log_{10}$ (CFU/mL). Source data are provided as a Source Data file.

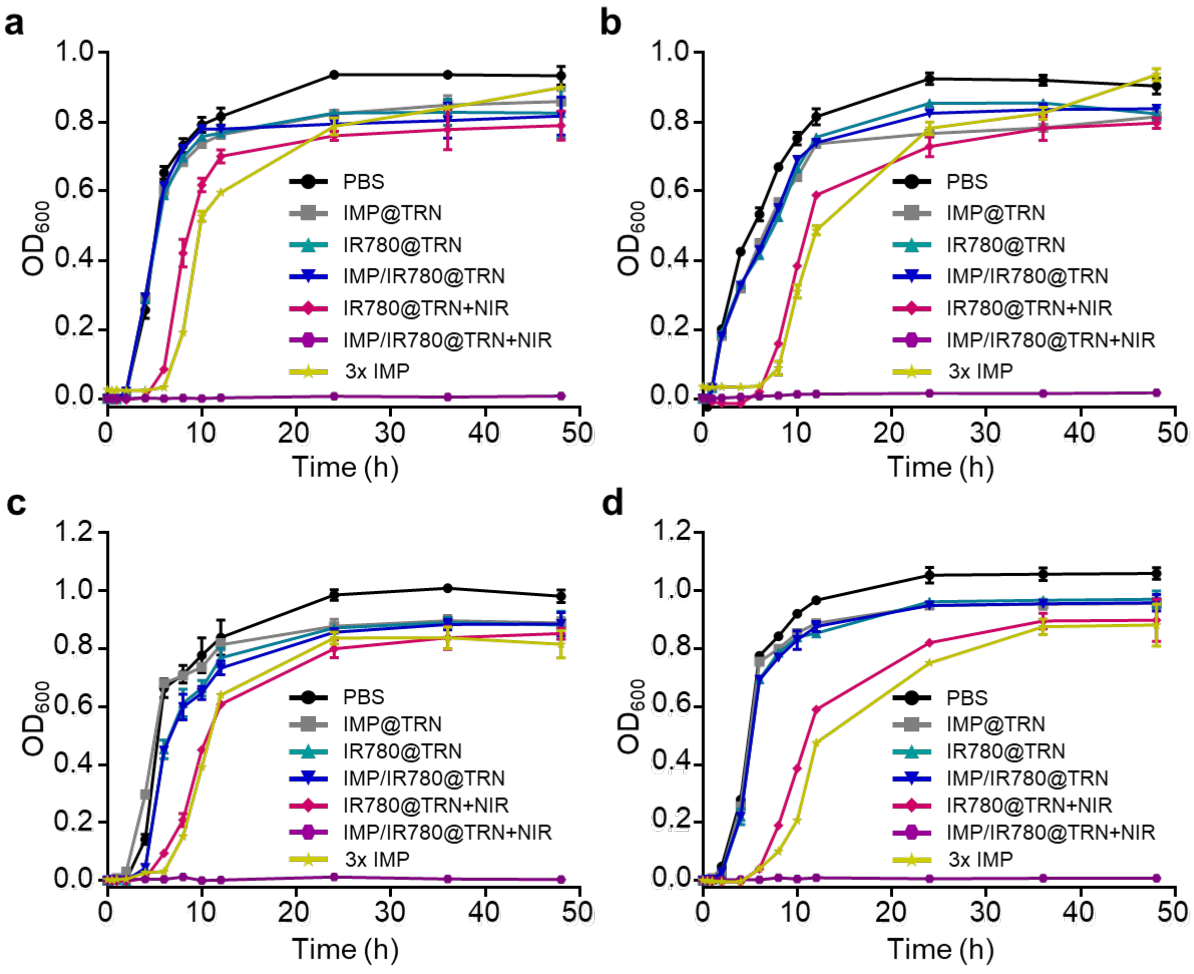

Supplementary Figure 8. Growth-inhibition assay in liquid medium. a *EC*. **b** *SA*. **c** MDREC. **d** MRSA. After treatment with different formulations, the bacterial suspensions were cultured at 37℃ and the optical density at 600 nm (OD_{600}) was measured at the certain points. Data are presented as mean ± s.d. (n=3). Source data are provided as a Source Data file.

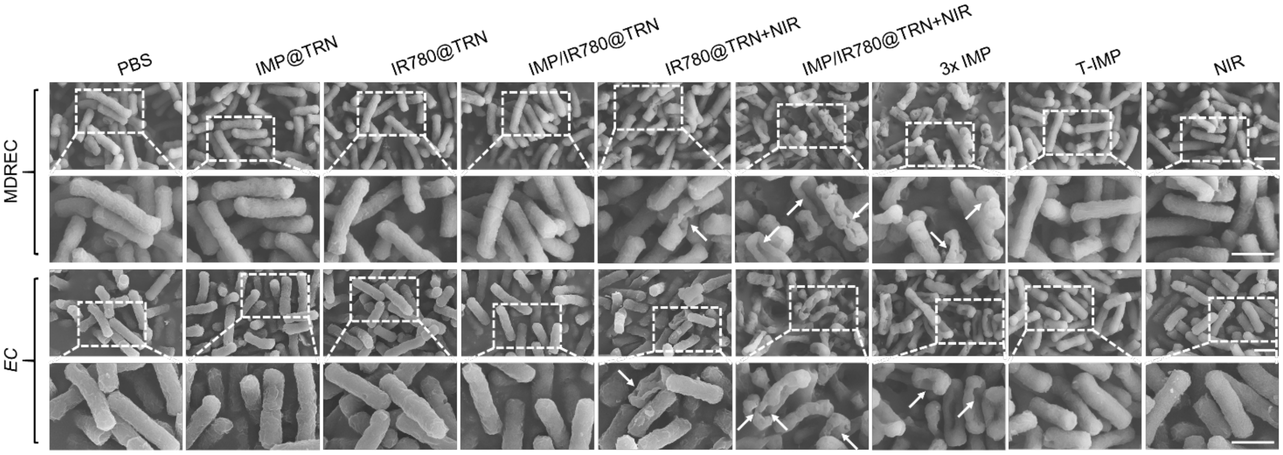

Supplementary Figure 9. SEM images of MDREC and *EC* treated with various conditions. White arrows denote the morphological damages in bacterial cell. Scale bars: 1 μm.

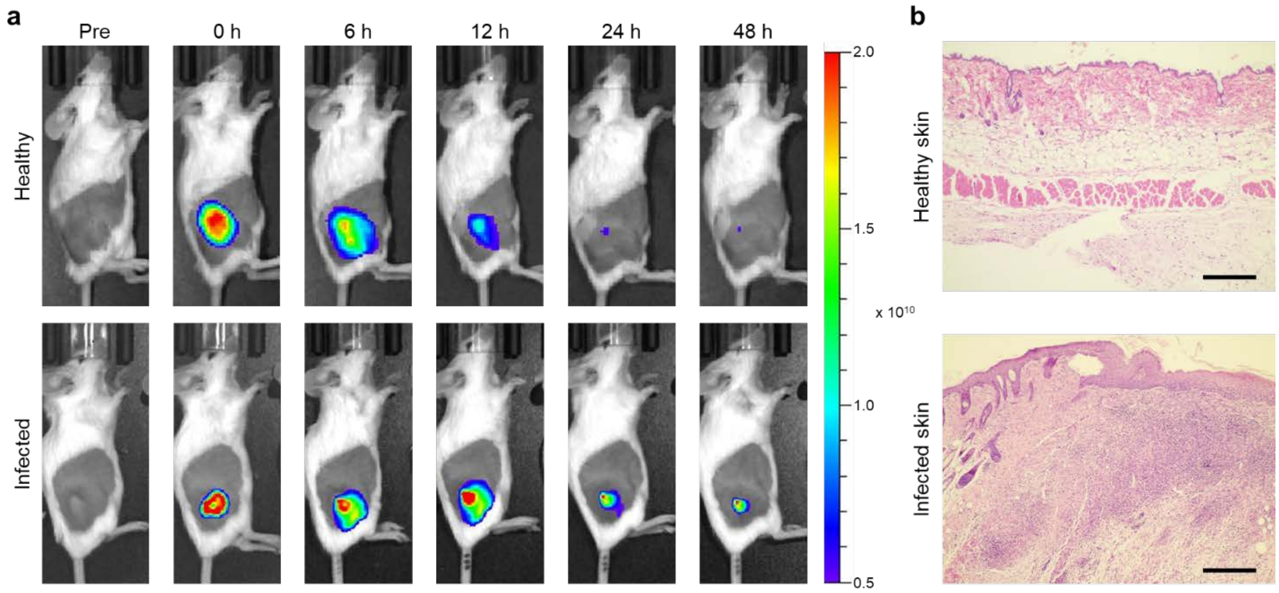

Supplementary Figure 10. The retention of the TRIDENT after the local injection *in vivo*. a Fluorescence images of skin obtained from healthy and infected mice. The TRIDENT can stay in infected area for a long time while drift away rapidly from the injection site in normal skin tissue. **b** Corresponding histological photomicrographs of heathy and infected skin tissue sections. Scale bars: 20 μm.

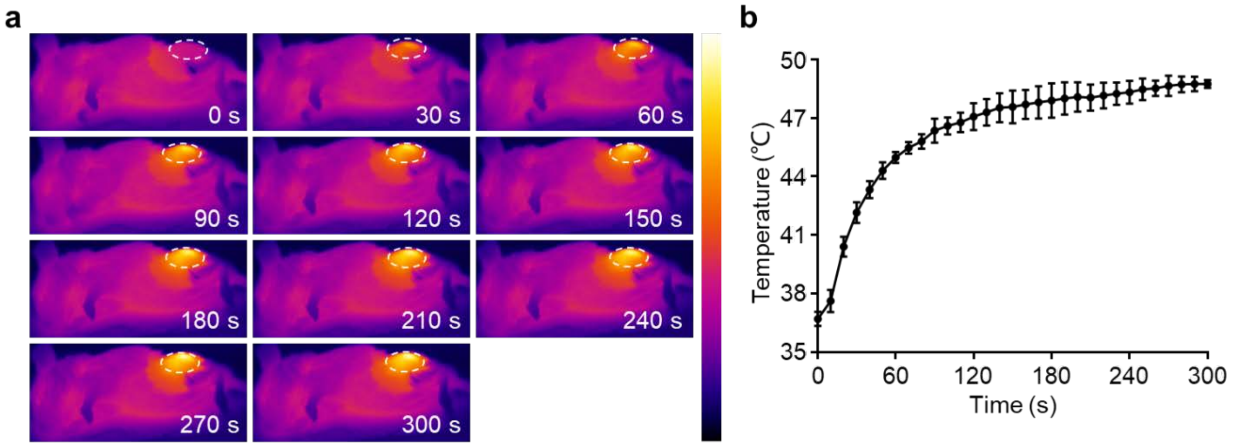

Supplementary Figure 11. *In vivo* photothermal experiments for the developed TRIDENT. a Thermal images of mice obtained from the test process. White dashed circles denote the infected skin. **b** Corresponding heating curve. Data are presented as mean ± s.d. (n=4). Source data are provided as a Source Data file.

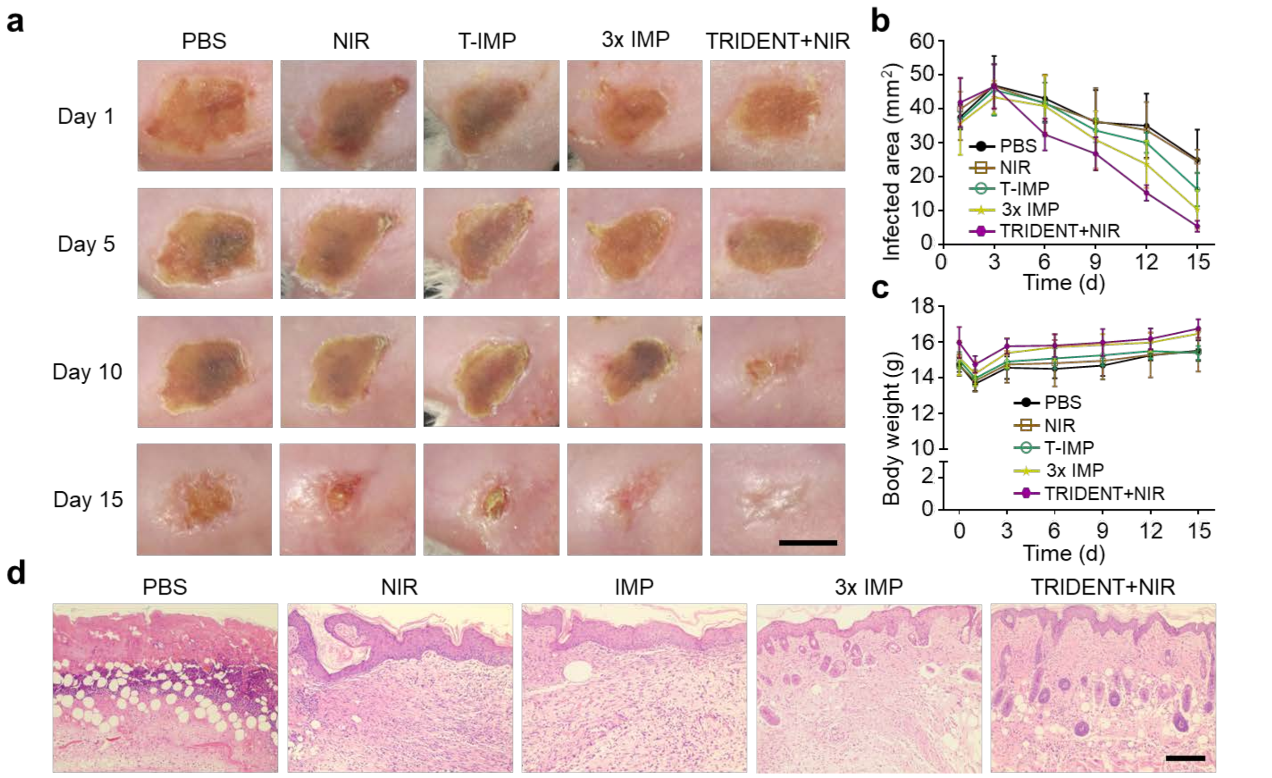

Supplementary Figure 12. Antibacterial activities of different ways in the MRSA-infected mice. a Photographs of the infected skin of mice during treatment within 15 days. Scale bar: 5 mm. **b** Size of the infected area and **c** body weights of the mice. Data are presented as mean ± s.d. (n=3). **d** Histological photomicrographs of skin tissue sections of infected mice after completion of the in vivo antibacterial activity experiment. Scale bar: 20 μm. Source data are provided as a Source Data file.

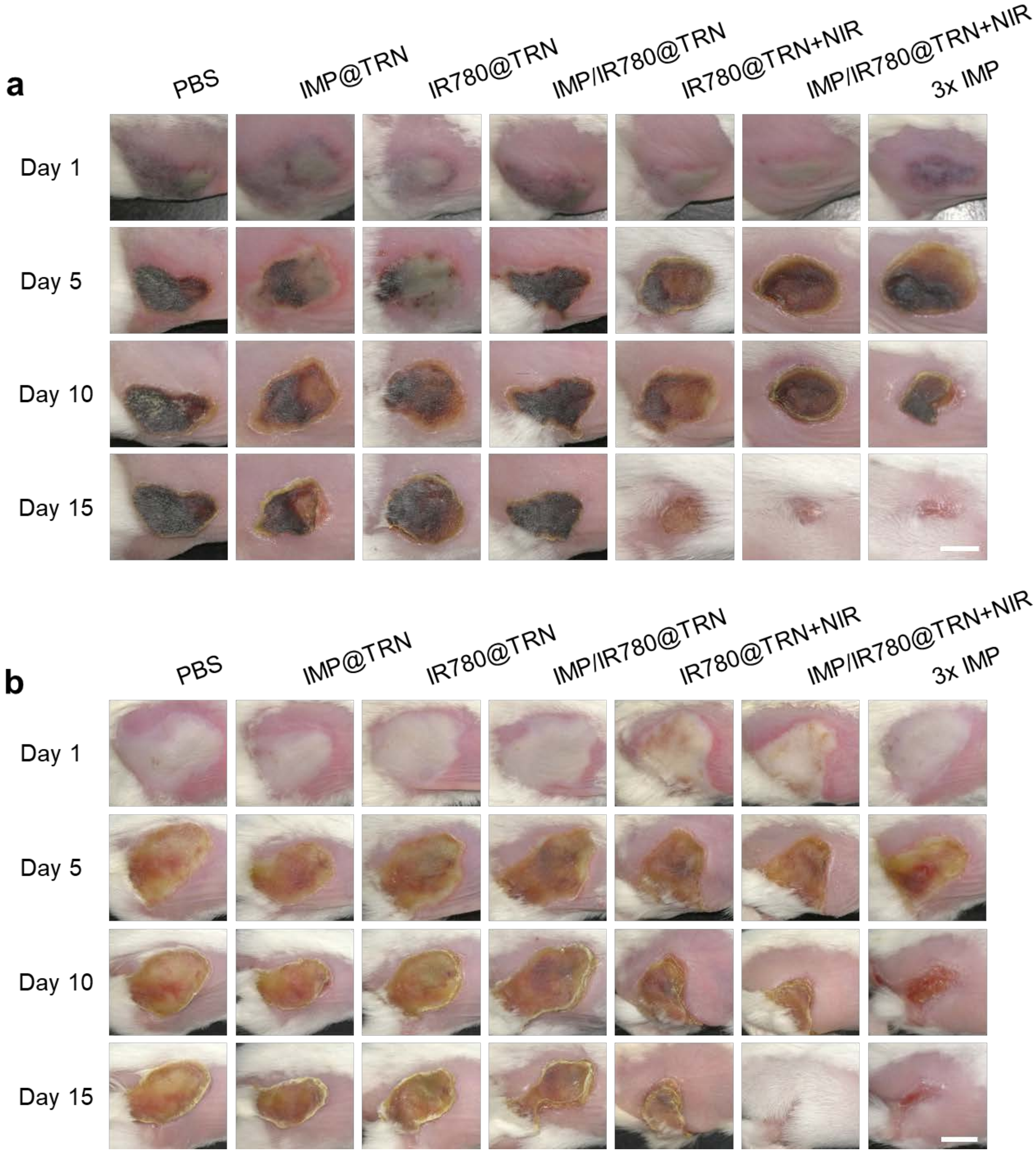

Supplementary Figure 13. Changes in infected skin treated with different formulations. a *EC*- and **b** *SA*-infected skin from mice during the treatment with different formulations for 15 days. Scale bars: 5 mm.

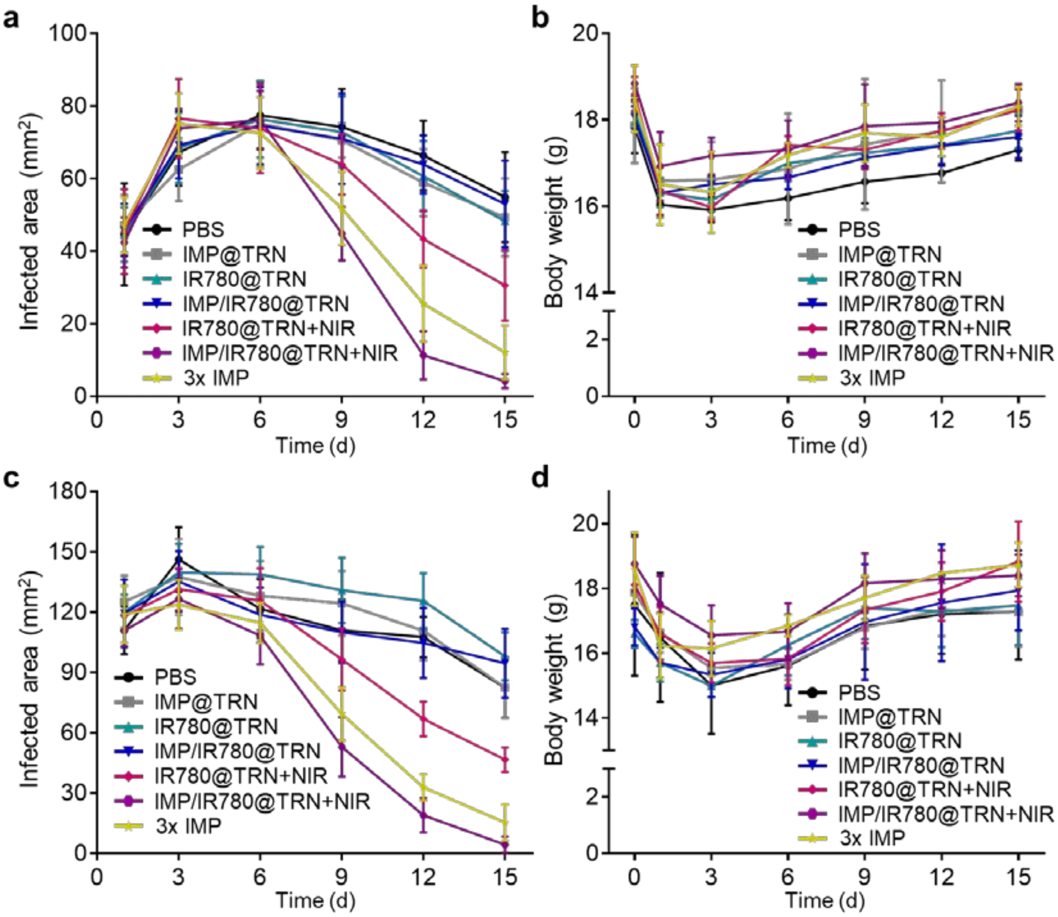

Supplementary Figure 14. Change of the infected area and body weight within the testing process. **a** Curves of infected area and **b** body weight obtained from *EC*-infected mice during treatment with the different formulations for 15 days. **c** Curves of infected area and **d** body weight obtained from *SA*-infected mice during treatment with the different formulations for 15 days. Data are presented as mean ± s.d. (n=3). Source data are provided as a Source Data file.

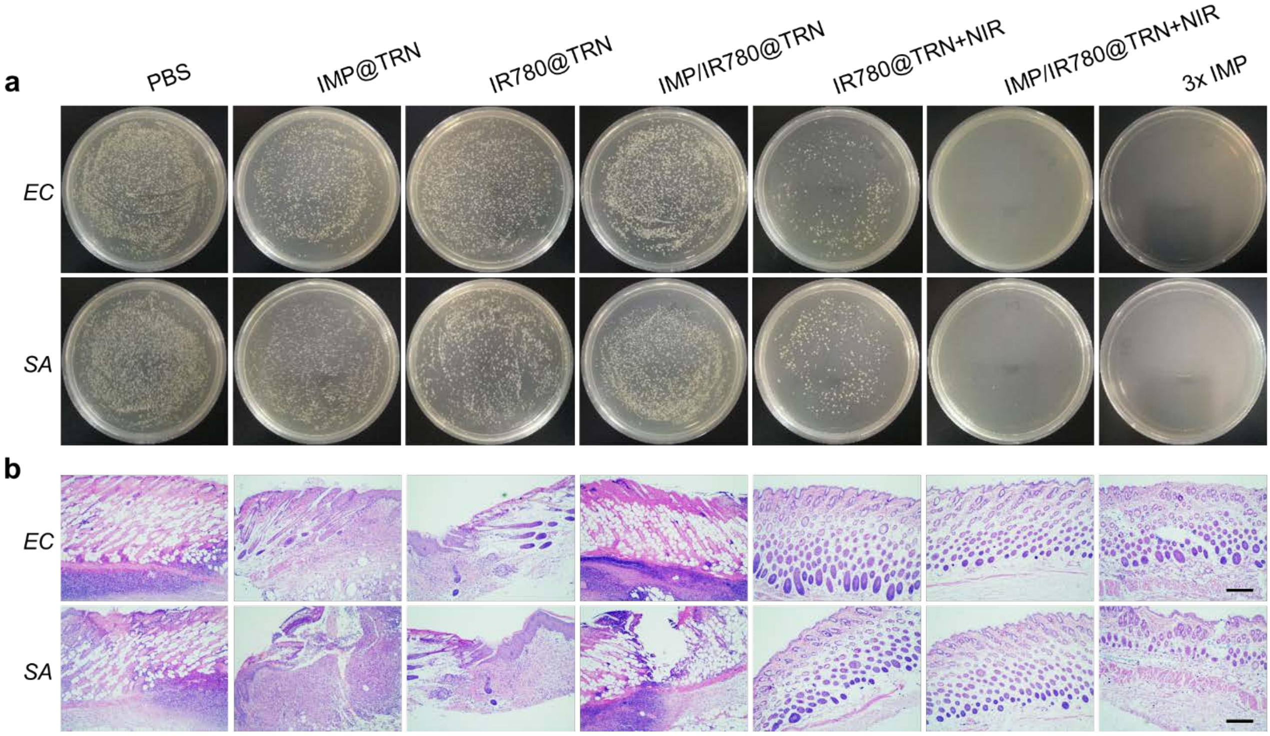

Supplementary Figure 15. Evaluation the rehabilitation of skin tissues. a Photographs of plated bacteria which were obtained from the infected tissues of mice in the different treatment groups. **b** Histological photomicrographs of skin tissue sections from infected mice in the different treatment groups. Scale bars: 20 μm.

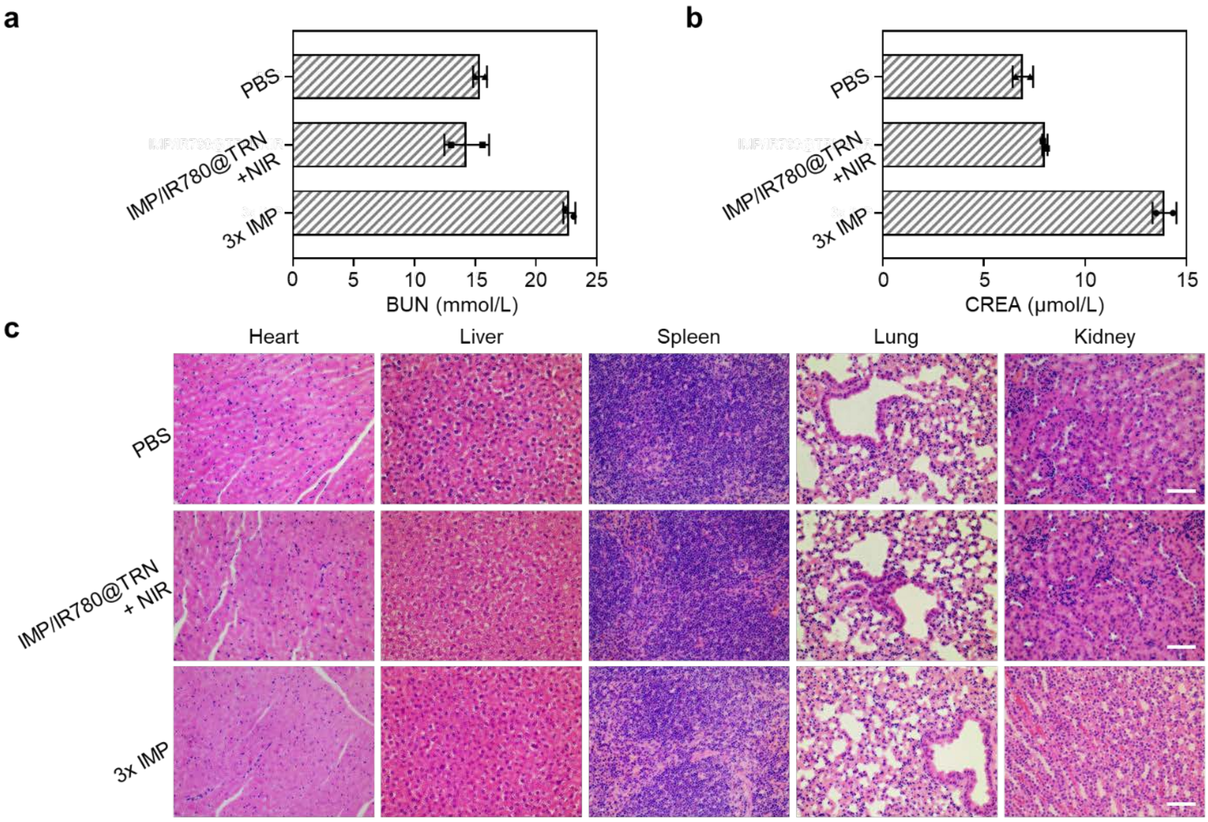

Supplementary Figure 16. *In vivo* biosafety evaluations of TRIDENT. a Biochemical analysis of blood urea nitrogen (BUN) and **b** creatinine (CREA) at completion of the treatment with PBS, TRIDENT+NIR or 3x IMP. PBS was administered as a control. Data are presented as mean ± s.d. (n=2). **c** Histological study of different organs (heart, liver, spleen, lung, and kidney) from mice at the end of treatment with PBS, IMP/IR780@TRN+NIR and 3x IMP. Scale bars: 50 μm. Source data are provided as a Source Data file.
