# Supplementary material for: Thermo-responsive triple-function nanotransporter for efficient chemo-photothermal therapy of multidrug-resistant bacterial infection
Source: Nat Commun. 2019 Sep 24;10:4336. doi: 10.1038/s41467-019-12313-3 (PMC6760232; doi:10.1038/s41467-019-12313-3)
Supplement: Supplementary file 3 — Reporting Summary [file 41467_2019_12313_MOESM3_ESM.pdf]

nature research

Corresponding author(s): Weisheng Guo, Yang Luo and Xing-Jie Liang

Last updated by author(s): Aug 26, 2019

Reporting Summary

Nature Research wishes to improve the reproducibility of the work that we publish. This form provides structure for consistency and transparency in reporting. For further information on Nature Research policies, see Authors & Referees and the Editorial Policy Checklist.

Statistics

For all statistical analyses, confirm that the following items are present in the figure legend, table legend, main text, or Methods section.

n/a	Confirmed	
☐	☒	The exact sample size (n) for each experimental group/condition, given as a discrete number and unit of measurement
☐	☒	A statement on whether measurements were taken from distinct samples or whether the same sample was measured repeatedly
☐	☒	The statistical test(s) used AND whether they are one- or two-sided<br>*Only common tests should be described solely by name; describe more complex techniques in the Methods section.*
☐	☒	A description of all covariates tested
☐	☒	A description of any assumptions or corrections, such as tests of normality and adjustment for multiple comparisons
☐	☒	A full description of the statistical parameters including central tendency (e.g. means) or other basic estimates (e.g. regression coefficient) AND variation (e.g. standard deviation) or associated estimates of uncertainty (e.g. confidence intervals)
☐	☒	For null hypothesis testing, the test statistic (e.g. F, t, r) with confidence intervals, effect sizes, degrees of freedom and P value noted<br>*Give P values as exact values whenever suitable.*
☒	☐	For Bayesian analysis, information on the choice of priors and Markov chain Monte Carlo settings
☒	☐	For hierarchical and complex designs, identification of the appropriate level for tests and full reporting of outcomes
☐	☒	Estimates of effect sizes (e.g. Cohen's d, Pearson's r), indicating how they were calculated

Our web collection on statistics for biologists contains articles on many of the points above.

Software and code

Policy information about availability of computer code

Data collection	Digital Micrograph3.7, ZEN 2010, Supcre G9
Data analysis	Statistical analyses were performed on Graphpad Prism or SPSS 17.0.

For manuscripts utilizing custom algorithms or software that are central to the research but not yet described in published literature, software must be made available to editors/reviewers. We strongly encourage code deposition in a community repository (e.g. GitHub). See the Nature Research guidelines for submitting code & software for further information.

Data

Policy information about availability of data

All manuscripts must include a data availability statement. This statement should provide the following information, where applicable:

- Accession codes, unique identifiers, or web links for publicly available datasets
- A list of figures that have associated raw data
- A description of any restrictions on data availability

The data that support the findings of this study are available within the paper and its Supplementary Information files, or are available from the corresponding authors upon reasonable request. The source data underlying Figs. 2c-e, 3a, 3c-f, 4b, c, 5b, c, 6d, e, 6g, h, 6j, 7a, b as well as Supplementary Figures 1, 3-8, 11-12, 14, 16 are provided as a Source Data file.

Field-specific reporting

Please select the one below that is the best fit for your research. If you are not sure, read the appropriate sections before making your selection.

☒ Life sciences ☐ Behavioural & social sciences ☐ Ecological, evolutionary & environmental sciences

For a reference copy of the document with all sections, see nature.com/documents/nr-reporting-summary-flat.pdf

Life sciences study design

All studies must disclose on these points even when the disclosure is negative.

Sample size	No effect size was predetermined, but sample sizes employed in this study are consistent with previously published works. For example, in vitro studies were repeated at least three times independently and in the in vivo experiments with 3-4 mice per group were performed.
Data exclusions	No animals and/or data were excluded.
Replication	All experiments were repeated and experimental findings were reproducible.
Randomization	The dosing groups were filled by randomly selecting from the same pool of animals for in vivo experiments.
Blinding	All the investigators were blinded to group allocation during data collection and analysis.

Reporting for specific materials, systems and methods

We require information from authors about some types of materials, experimental systems and methods used in many studies. Here, indicate whether each material, system or method listed is relevant to your study. If you are not sure if a list item applies to your research, read the appropriate section before selecting a response.

Materials & experimental systems

n/a	Involved in the study	
☐	☒	Antibodies
☐	☒	Eukaryotic cell lines
☒	☐	Palaeontology
☐	☒	Animals and other organisms
☒	☐	Human research participants
☒	☐	Clinical data

Methods

n/a	Involved in the study	
☒	☐	ChIP-seq
☒	☐	Flow cytometry
☒	☐	MRI-based neuroimaging

Antibodies

Antibodies used	The following primary antibodies were used for enzyme-linked immunosorbent assay. They are listed as antibody first, followed by supplier, catalognumber and clone/lot number as applicable.<br>1). Anti-PCT, Andy Gene, cat. no. M0218
Validation	All antibodies were verified by the supplier and each lot has been quality tested.

Eukaryotic cell lines

Policy information about cell lines

Cell line source(s)	3T3 and HUVEC cells were purchased from the Institute of Basic Medical Sciences, Chinese Academy of Medical Sciences.
Authentication	A short tandem repeat DNA profiling method was used to authenticate the cell lines and the results were compared with reference database. Moreover, no mycoplasma contamination was detected in the above cell lines.
Mycoplasma contamination	All cell lines were tested for mycoplasma contamination. No mycoplasma contamination was found.
Commonly misidentified lines (See ICLAC register)	No. 3T3 and HUVEC cell lines are not listed in the database.

Animals and other organisms

Policy information about studies involving animals; ARRIVE guidelines recommended for reporting animal research

Laboratory animals	Balb/c mice (female, 6-8 weeks) were purchased from Beijing Charles River Company (Beijing, China).
Wild animals	No wild animals were used in this study.
Field-collected samples	The study did not involve samples collected from field.
Ethics oversight	All animal experiment protocols were reviewed and approved by the Institutional Animal Care and Use Committee of National Center for Nanoscience and Technology and complied with all relevant ethical regulations.

Note that full information on the approval of the study protocol must also be provided in the manuscript.
